# Nature-Inspired Chemical Reaction Optimisation Algorithms

**DOI:** 10.1007/s12559-017-9485-1

**Published:** 2017-06-17

**Authors:** Nazmul Siddique, Hojjat Adeli

**Affiliations:** 1 School of Computing and Intelligent Systems, University of Ulster, Northland Road, Londonderry, BT48 7JL UK; 2 College of Engineering, The Ohio State University, 470 Hitchcock Hall, 2070 Neil Avenue, Columbus, OH 43210 USA

**Keywords:** Nature-inspired computing, Biologically inspired algorithm, Physics inspired algorithms, Chemical reaction optimisation

## Abstract

Nature-inspired meta-heuristic algorithms have dominated the scientific literature in the areas of machine learning and cognitive computing paradigm in the last three decades. Chemical reaction optimisation (CRO) is a population-based meta-heuristic algorithm based on the principles of chemical reaction. A chemical reaction is seen as a process of transforming the reactants (or molecules) through a sequence of reactions into products. This process of transformation is implemented in the CRO algorithm to solve optimisation problems. This article starts with an overview of the chemical reactions and how it is applied to the optimisation problem. A review of CRO and its variants is presented in the paper. Guidelines from the literature on the effective choice of CRO parameters for solution of optimisation problems are summarised.

## Introduction

Machine learning is one of the most important platforms for cognitive computation paradigm. The main constituents of machine learning are the meta-heuristic algorithms. Nature has always been an inspiration and source for scientific invention. Scientists have been striving for understanding the laws of nature and developing methods and computer algorithms for real-life problems. The development of three mainstream disciplines of the natural sciences, biology, physics, and chemistry have created novel problem solving paradigms [1, 2]. Biology-inspired algorithms have been in use since 1960. They include genetic algorithms [3], genetic programming [4], evolutionary computation [5], and particle swarm optimisation [6]. Physics inspired algorithms have been the subject of significant research in the last three decades [7]. They include simulated annealing [8, 9], gravitational search algorithm [10], harmony search algorithm [11], central force optimization [12], water drop algorithm [13], and spiral dynamics algorithm [14]. The chemical reaction metaphor can also be exploited for developing meta-heuristic algorithms by encoding appropriate information into molecule-like elements and performing a set of chemical reaction-like operations onto them to obtain certain kind of derivative information suitable for optimisation problems. Chemical reaction optimisation (CRO) algorithm is a recent search and optimisation algorithm inspired by chemistry, which is equally promising like biology and physics inspired algorithms.

Chemistry is the field of science that studies the chemical properties of matter and its structure. Chemical reactions break chemical bonds into molecules and form new bonds using molecules participating in reaction [15]. Energy is required for breaking chemical bonds into several molecules. Also energy is released when new bonds are formed combining several molecules. Thus, a chemical reaction is seen as a process of transformation of a set of molecules participating in a chemical reaction into a set of products with different properties. There are two types of chemical reactions, namely, uni-molecular and multi-molecular elementary reactions absorbing or releasing different level of energies during the reactions [16]. Chemical reactions uphold the laws of thermodynamics. Every chemical reaction stabilises at equilibrium, a state determined by the minimum free energy, also called Gibbs free energy [17]. The Gibbs free energy is a chemical potential that depends on the temperature, pressure, and materials involved and tends to reach its minimum at equilibrium.

A molecule consists of several atoms. The type of atoms, bond length, angle, and torsion (twisting of the structure) define the distinct structure of the molecule. Two molecules can be different even with the same set of atoms due to the difference in their structures. In other words, the structure represents the relationship between atoms in a molecule. In order to undergo a chemical reaction, molecules must acquire the necessary energy to be activated. Chemical bonds are source of energy. The energy of a molecule can be of two types: potential energy and kinetic energy. Potential energy is denoted by PE. PE is the energy that a molecule contains in the structure. In a chemical reaction (e.g. exothermic reaction), chemical bonds break and new bond are formed. That is, during the reaction molecules transform from structure of higher PE into structure of lower PE with release of energy. Kinetic energy is denoted by KE. Molecules need to collide for the chemical reaction to happen. Collision between molecules provides the KE needed to break the bonds. Sometimes there is not enough KE for collisions to happen. In such a case, energy is provided in the form of heat. Heat rises the temperature which is a measure of the average KE. Heating causes KE to increase to the required level for breaking the bonds.

If *x* = {*x* ^1^, *x* ^2^,  ⋯ , *x* ^*n*^} is a molecule with *x* ^*k*^, *k* = 1 ,  ⋯  , *n* atoms of a certain structure, then the molecule tends to change from *x* to a new molecule *x* ^′^ by changing its structure during a chemical reaction only if the following energy condition is satisfied.

1 $$ \mathrm{PE}(x)\ge \mathrm{PE}\left({x}^{\prime}\right) $$

If energy Condition (1) is not satisfied, the molecules need higher energy for the reactions to happen. KE is added to achieve the required higher energy level for the reactions to occur and change to new molecules. This must satisfy the following energy condition.

2 $$ \mathrm{PE}(x)+\mathrm{KE}(x)\ge \mathrm{PE}\left({x}^{\prime}\right) $$

A molecule with higher KE has a higher possibility of transforming into a new structure with higher PE. A reactant (generally a molecule or a compound) with high energy is unstable and tends to have a reaction when it comes in contact with another molecule and goes through a sequence of elementary reaction phases dissipating energy. The process is called reaction mechanism. A chemical reaction may take more than one reaction path ensuring the maximum amounts of desired products and the minimum amounts of undesired products. The actual course of any reaction is determined by the least energy requirement leading to an optimal reaction mechanism. At the final stage of reaction, products produced will have low-energy level and stable state. The final state is considered an optimal and stable state of the chemical reaction maintaining an optimal reaction mechanism.

Lam and Li [18] proposed the Chemical Reaction Optimization (CRO) algorithm inspired by chemical reactions. This paper presents the CRO algorithm, different variants of CRO, hybrids of CRO with other meta-heuristic methods and their applications in different domains. The rest of the paper is organised as follows: Section “Chemical Reaction Operations in CRO” presents the background on the chemical reaction operations used in CRO. Section “Implementation of CRO Operation” describes the implementation of the CRO operators. Section “Chemical Reaction Optimisation Algorithm” presents the CRO algorithm. Variants and hybrid CRO algorithms are presented in Section “Variants of CRO”. Section “Conclusions” concludes the paper with some comments on the future directions of CRO algorithm.

## Chemical Reaction Operations in CRO

Molecules with different levels of energy take part in chemical reactions, undergo a sequence of elementary reactions and are transformed into products with minimum energy. The elementary reactions are the operators of CRO. These reactions are grouped into uni-molecular and multi-molecular reactions, classified into four types:

- Uni-molecular reactions
  (i) On-wall ineffective collision
  (ii) Decomposition
- Multi-molecular reactions
  (iii) Inter-molecular ineffective collision
  (iv) Synthesis

### On-Wall Ineffective Collision Operation

**Fig. 1:**
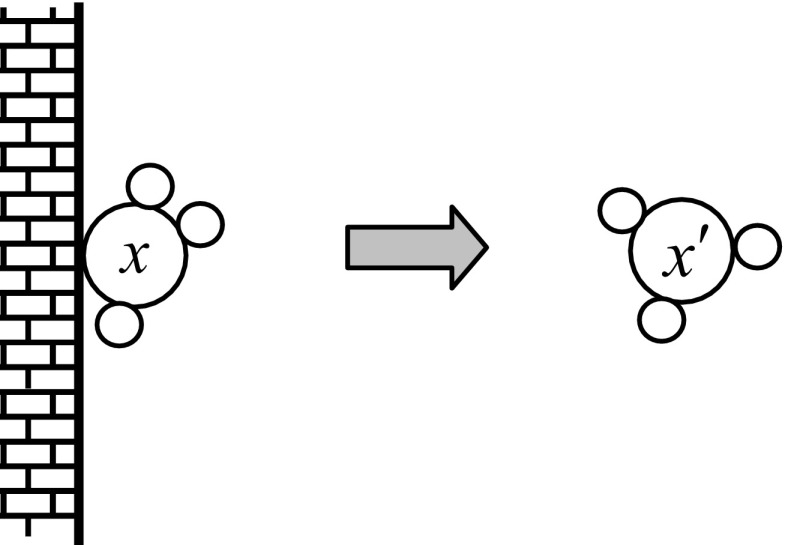
Illustration of on-wall ineffective collision operator. The structure of molecule is shown by the *big circles* with attached *small circles* around representing the change in the structure. A molecule hits on the wall and bounces back with perturbed structure. The perturbed structure is shown with changed positions of the *small circles*

Only one molecule is involved in this type of operation. The molecule does not take part in chemical reaction with another molecule. A molecule *x* hits on the wall and bounces back resulting in a change in PE and KE. The new value is denoted as *x* ^′^. It is, therefore, called on-wall ineffective collision. The on-wall ineffective collision of a molecule is illustrated in Fig. 1. The change of the molecule is described by

3 $$ {x}^{\prime }= x+\Delta $$

where Δ is a perturbation of the molecular structure caused by the collision. The perturbation Δ can be modelled as probability distribution over a finite interval, e.g. Gaussian, Cauchy, lognormal, and exponential distribution. Due to the change in molecule, PE(*x*) changes to PE(*x* ^′^) and KE(*x*) changes to KE(*x* ^′^), which must satisfy the energy Eq. (2). Otherwise, a change in the molecule *x* will not happen without release (or loss) of some amount of kinetic energy KE(*x*). According to the law of conservation of energy, energy cannot be destroyed. Therefore, the released energy KE(*x* ^′^) is stored in an energy buffer called central buffer after the reaction. In CRO, the released energy KE(*x* ^′^) is modelled using a random number *ρ*_1_ ∈ [KE_LossRate_, 1], where KE_LossRate_ is a parameter of the chemical reaction and represents the maximum percentage of KE lost in the environment at a time. (1 − *ρ*_1_) is the fraction of KE that is lost in the environment when the molecule hits the wall. This lost energy is stored in the central buffer. KE(*x* ^′^) and energy buffer update are described by

4 $$ \mathrm{KE}\left({x}^{\prime}\right)=\left[\mathrm{PE}(x)-\mathrm{PE}\left({x}^{\prime}\right)+\mathrm{KE}(x)\right]\times {\rho}_1 $$

5 $$ \mathrm{buffer}=\mathrm{buffer}+\left[\mathrm{PE}(x)-\mathrm{PE}\left({x}^{\prime}\right)+\mathrm{KE}(x)\right]\times \left(1-{\rho}_1\right) $$

### Decomposition Operation

**Fig. 2:**
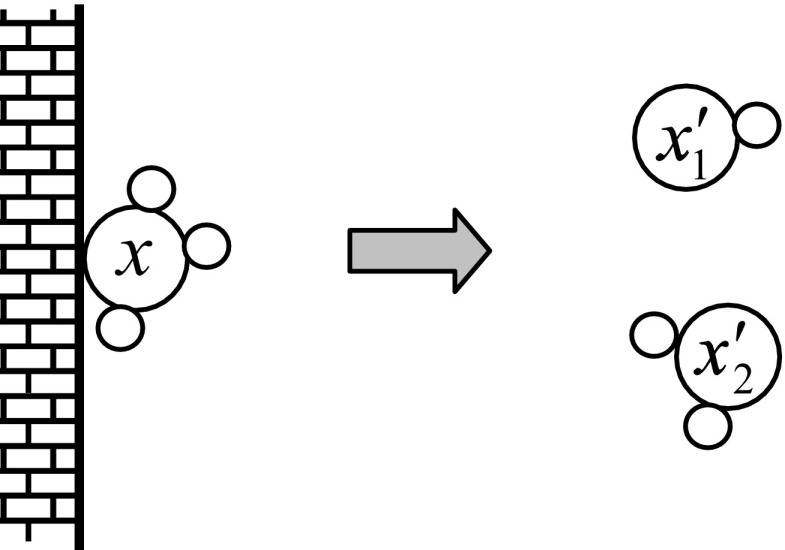
Illustration of decomposition operation. A molecule hits on the wall and decomposes into two molecules with change in structures

One molecule is involved in this type of operation. The molecule does not take part in chemical reaction with another molecule. A molecule *x* hits the wall and decomposes into two molecules *x*_1_ and *x*_2_. The molecule can also decompose into more than two molecules. The decomposition of molecule *x* into molecules *x*_1_ and *x*_2_ is illustrated in Fig. 2. Due to the change in the molecule structure, PE(*x*) changes to PE(*x*_1_) and PE(*x*_2_) and KE(*x*) changes to KE(*x*_1_) and KE(*x*_2_) which must satisfy the energy conditions described by

6 $$ \mathrm{PE}(x)+\mathrm{KE}(x)\ge \mathrm{PE}\left({x}_1^{\prime}\right)+\mathrm{PE}\left({x}_2^{\prime}\right) $$

7 $$ \mathrm{KE}\left({x}_1^{\prime}\right)=\left[\mathrm{PE}(x)+\mathrm{KE}(x)-\mathrm{PE}\left({x}_1^{\prime}\right)-\mathrm{PE}\left({x}_2^{\prime}\right)\right]\times {\rho}_1 $$

8 $$ \mathrm{KE}\left({x}_2^{\prime}\right)=\left[\mathrm{PE}(x)+\mathrm{KE}(x)-\mathrm{PE}\left({x}_1^{\prime}\right)-\mathrm{PE}\left({x}_2^{\prime}\right)\right]\times \left(1-{\rho}_1\right) $$

where *ρ*_1_ ∈ [0, 1] is a random number representing the released energy.

Sometimes molecule *x* does not have enough energy meaning that Condition (6) does not hold for the reaction to happen and decompose into *x*_1_ and *x*_2_. It can only happen when energy KE(*x*) is large enough. According to the law of conservation of energy, energy cannot be created. The extra energy comes from the central energy buffer to decompose the molecule. The process is described by the following modified conditions:

9 $$ \mathrm{PE}(x)+\mathrm{KE}(x)+\mathrm{buffer}\ge \mathrm{PE}\left({x}_1^{\prime}\right)+\mathrm{PE}\left({x}_2^{\prime}\right) $$

10 $$ \mathrm{KE}\left({x}_1^{\prime}\right)=\left[\left\{\mathrm{PE}(x)+\mathrm{KE}(x)-\mathrm{PE}\left({x}_1^{\prime}\right)-\mathrm{PE}\left({x}_2^{\prime}\right)\right\}+\mathrm{buffer}\right]\times \left({\rho}_1\times {\rho}_2\right) $$

11 $$ \mathrm{KE}\left({x}_2^{\prime}\right)=\left[\left\{\mathrm{PE}(x)+\mathrm{KE}(x)-\mathrm{PE}\left({x}_1^{\prime}\right)-\mathrm{PE}\left({x}_2^{\prime}\right)\right\}+\mathrm{buffer}\right]\times \left({\rho}_3\times {\rho}_4\right) $$

where {*ρ*_1_, *ρ*_2_, *ρ*_3_, *ρ*_4_} ∈ [0, 1] are random numbers. To ensure a small amount of energy for $\mathrm{KE}\left({x}_1^{\prime}\right)$ and $\mathrm{KE}\left({x}_2^{\prime}\right)$ from the buffer, multiplication of two random numbers {*ρ*_1_ × *ρ*_2_} and {*ρ*_3_ × *ρ*_4_} are used. The energy buffer update is described by

12 $$ \mathrm{buffer}=\mathrm{buffer}+\left[\mathrm{PE}(x)+\mathrm{KE}(x)-\mathrm{PE}\left({x}_1^{\prime}\right)-\mathrm{PE}\left({x}_2^{\prime}\right)\right]-\mathrm{KE}\left({x}_1^{\prime}\right)-\mathrm{KE}\left({x}_2^{\prime}\right) $$

If Conditions in (6) and (9) do not hold, the decomposition will not take place.

### Inter-Molecular Ineffective Collision Operation

**Fig. 3:**
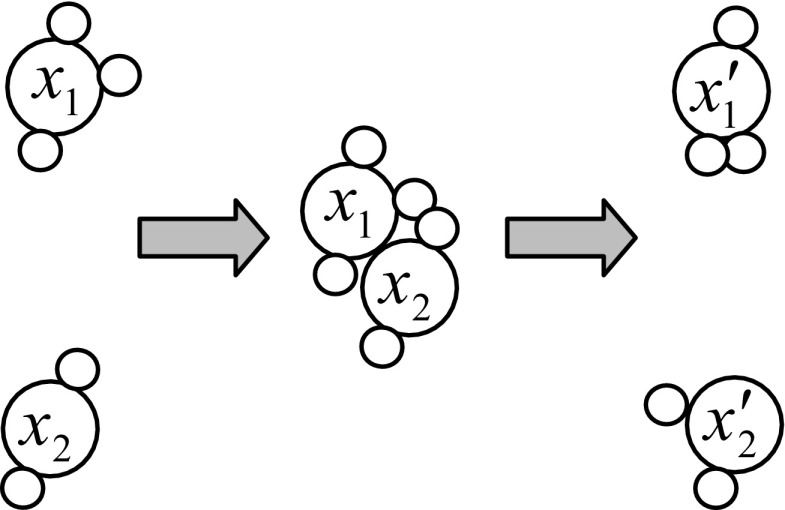
Illustration of inter-molecular ineffective collision operation. Two molecules *x*_1_ and *x*_2_ collide against each other and change to new molecules ${x}_1^{\prime }$ and ${x}_2^1$

Two molecules are involved in this type of operation. A molecule *x*_1_ collides with another molecule *x*_2_ and the two molecules *x*_1_ and *x*_2_ are perturbed and change to ${x}_1^{\prime }$ and ${x}_2^{\prime }$. Thus, the collision causes change in potential energies {PE(*x*_1_), PE(*x*_2_)} and kinetic energies {KE(*x*_1_), KE(*x*_2_)}. The inter-molecular collision operation is illustrated in Fig. 3. The potential energies {PE(*x*_1_), PE(*x*_2_)} change to {$\mathrm{PE}\left({x}_1^{\prime}\right)$, $\mathrm{PE}\left({x}_2^{\prime}\right)$} and the kinetic energies {KE(*x*_1_),KE(*x*_2_)} change to {$\mathrm{KE}\left({x}_1^{\prime}\right)$, $\mathrm{KE}\left({x}_2^{\prime}\right)$}, respectively. The inter-molecular ineffective collision must hold the energy conditions:

13 $$ \mathrm{PE}\left({x}_1\right)+\mathrm{PE}\left({x}_2\right)+\mathrm{KE}\left({x}_1\right)+\mathrm{KE}\left({x}_2\right)\ge \mathrm{PE}\left({x}_1^{\prime}\right)+\mathrm{PE}\left({x}_2^{\prime}\right) $$

14 $$ \mathrm{KE}\left({x}_1^{\prime}\right)=\left[\mathrm{PE}\left({x}_1\right)+\mathrm{PE}\left({x}_2\right)+\mathrm{KE}\left({x}_1\right)+\mathrm{KE}\left({x}_2\right)-\mathrm{PE}\left({x}_1^{\prime}\right)-\mathrm{PE}\left({x}_2^{\prime}\right)\right]\times {\rho}_1 $$

15 $$ \mathrm{KE}\left({x}_2^{\prime}\right)=\left[\mathrm{PE}\left({x}_1\right)+\mathrm{PE}\left({x}_2\right)+\mathrm{KE}\left({x}_1\right)+\mathrm{KE}\left({x}_2\right)-\mathrm{PE}\left({x}_1^{\prime}\right)-\mathrm{PE}\left({x}_2^{\prime}\right)\right]\times \left(1-{\rho}_1\right) $$

If Condition (13) does not hold, the reaction will not take place.

### Synthesis Operation

**Fig. 4:**
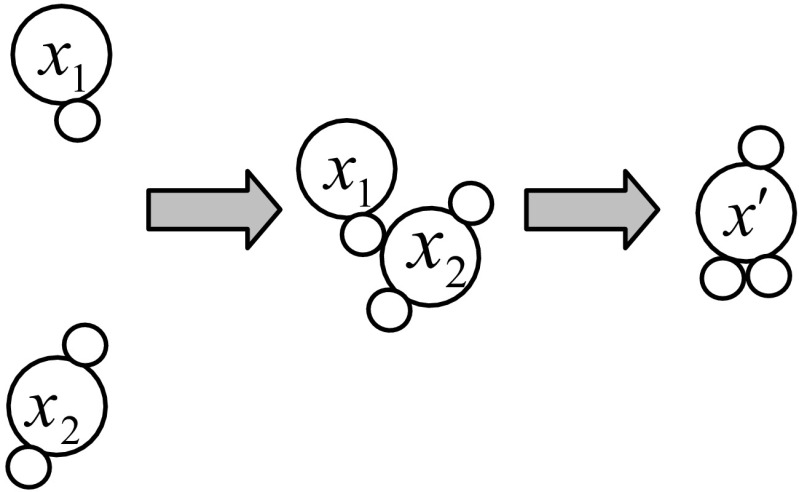
Illustration of synthesis operator. Two molecules *x*_1_ and *x*_2_ collide together and fuse into one molecule *x* ^′^

Two or more molecules are involved in this type of operation. Two molecules *x*_1_ and *x*_2_ collide together and fuse into a molecule *x* ^′^. Fusion releases a large amount of energy. Therefore, a large energy change occurs during the synthesis operation. The synthesis operation is illustrated in Fig. 4. The potential energies PE(*x*_1_) and PE(*x*_2_) change to PE(*x* ^′^) and the kinetic energies KE(*x*_1_) and KE(*x*_2_) change to KE(*x* ^′^). The energy balance of synthesis operation must hold the energy conditions:

16 $$ \mathrm{PE}\left({x}_1\right)+\mathrm{PE}\left({x}_2\right)+\mathrm{KE}\left({x}_1\right)+\mathrm{KE}\left({x}_2\right)\ge \mathrm{PE}\left({x}^{\prime}\right) $$

17 $$ \mathrm{KE}\left({x}^{\prime}\right)=\left[\mathrm{PE}\left({x}_1\right)+\mathrm{PE}\left({x}_2\right)+\mathrm{KE}\left({x}_1\right)+\mathrm{KE}\left({x}_2\right)-\mathrm{PE}\left({x}^{\prime}\right)\right] $$

If Condition (16) does not hold, the synthesis reaction will not take place.

In a chemical reaction process, a population of reactants with high-energy level undergoes a sequence of elementary chemical reactions, transforms through different energy levels and produces certain products with new molecular structures of low energy and stable states at the final stage. Every chemical reaction seeks to achieve equilibrium after which no further reactions take place. The chemical reaction process continues until it satisfies the aforementioned energy equations. The process of the chemical reaction is seen as an optimisation process where the set of parameters to be optimised are reactants that take part in the transformation process.

## Implementation of CRO Operation

CRO is a population-based optimisation algorithm. Chemical reaction operations discussed in earlier section are the inspiration behind the operators of CRO. Four types of operators are applied to the population of solutions. The total number of solutions kept by the algorithm may change from time to time as the decomposition and synthesis operators increase and decrease the number of molecules in the reaction pool, respectively. The computational implementation of these four operators is discussed in this section.

### Ineffective Collision Operator

Some small change occurs in the molecular attributes during this operation and the molecule *x* obtains a new structure *x* ^′^ in the neighbourhood of *x* which is expressed as:

18 $$ {x}^{\prime }= N(x) $$

where *N*(⋅) is the neighbourhood operator.

Yu et al. [19] used a neighbourhood operator *N*(*x*) to generate a new solution *x* ^′^ by perturbing one element of *x* chosen randomly. The perturbation is done by adding a Gaussian perturbation *ρ*(*m*, *σ*) to the randomly chosen element *m*, where *m* is the mean and *σ* is the variance. *σ* is a parameter and chosen arbitrarily. The neighbourhood operator is implemented using the following pseudo-code.
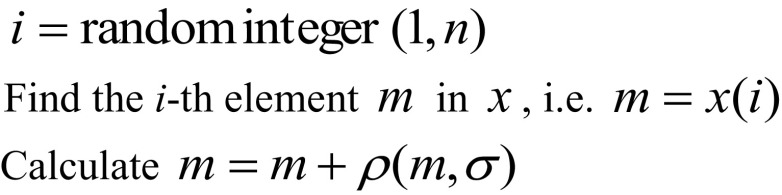


### Decomposition Operator

Decomposition operator breaks a molecule into two or more molecules. Firstly, the solution *x* is copied onto *x*_1_ and *x*_2_. Then half of the variables (i.e. *n/*2 variables where *n* is the total number of variables) of the solution *x* are perturbed by adding random variations and creating new solutions. The following pseudo-code is used to implement the decomposition operator.
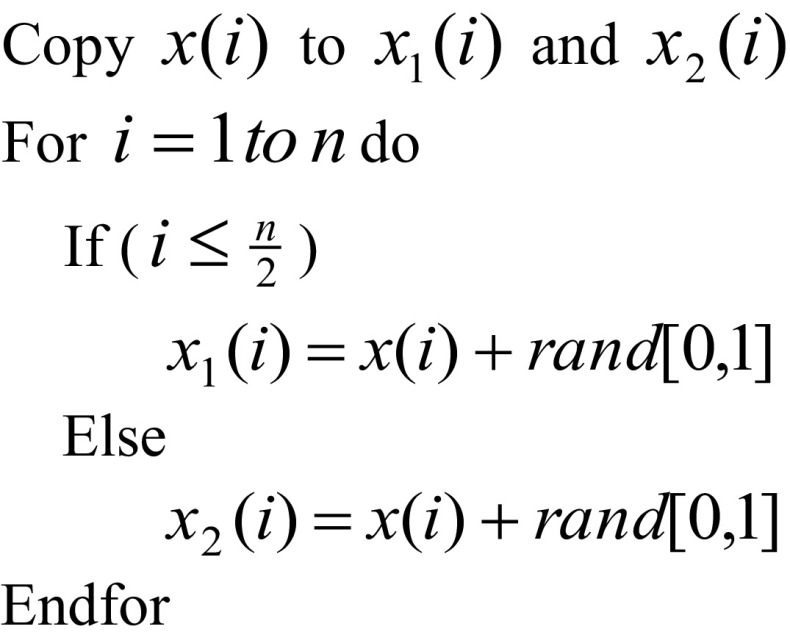


### Inter-Molecular Ineffective Collision Operator

Two molecules *x*_1_ and *x*_2_ collide with each other and change to new molecules ${x}_1^{\prime }$ and ${x}_2^{\prime }$ in the neighbourhood of *x*_1_ and *x*_2_. ${x}_1^{\prime }$ and ${x}_2^{\prime }$ are expressed as.

19 $$ \left\{\begin{array}{c}\hfill {x}_1^{\prime }= N\left({x}_1\right)\hfill \\ {}\hfill {x}_2^{\prime }= N\left({x}_2\right)\hfill \end{array}\right. $$

Implementation of the neighbourhood operator is presented in Section “Ineffective collision operator”.

### Synthesis Operator

Synthesis operator combines multiple molecules into one. Two solutions *x*_1_ and *x*_2_ are combined by applying a ‘probabilistic select’ to implement synthesis into a new solution *x* ^′^. Each component of *x* ^′^ in the same position is chosen either from *x*_1_ or *x*_2_ randomly. The following pseudo-code is used to implement the synthesis operator.
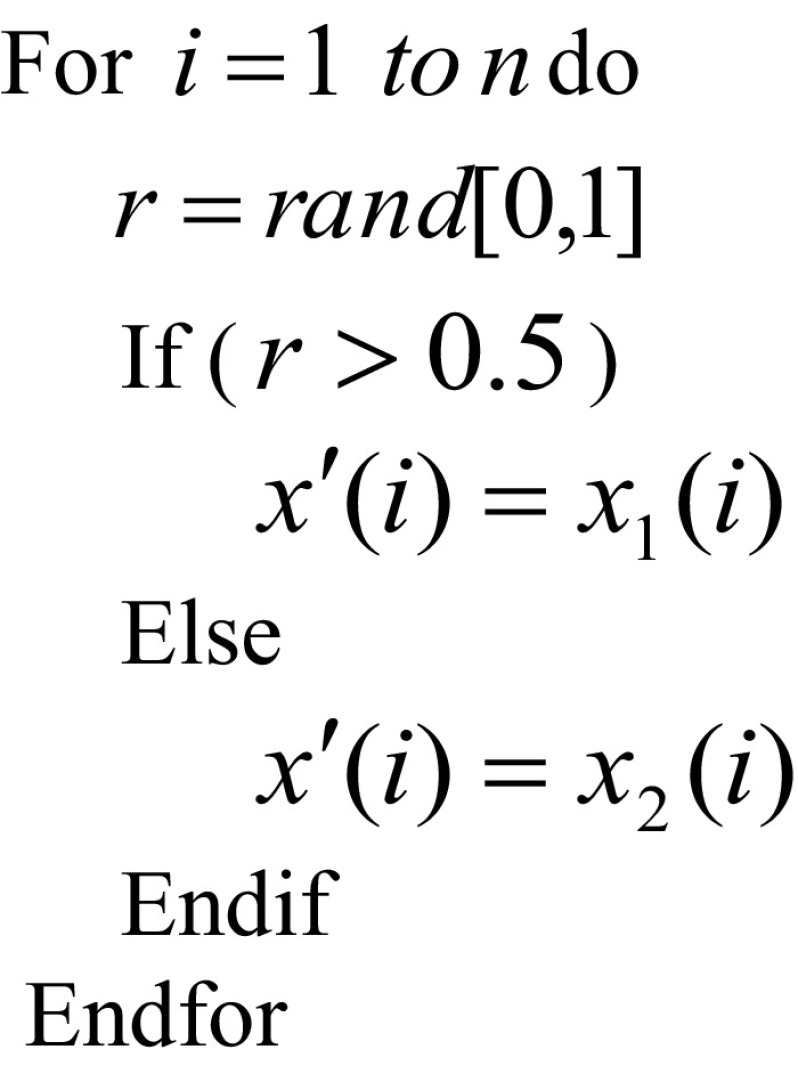


The effect of synthesis operator is similar to the recombination (or crossover) operation used by other evolutionary algorithms (EA)^1^ [21, 22].

In CRO, decomposition and synthesis are exploration mechanisms providing the effect of global search while ineffective collision and inter-molecular ineffective collision are exploitation mechanisms providing the effect of local search.

## Chemical Reaction Optimisation Algorithm

In terms of optimisation, a molecule with its atoms in a particular structure can be thought of a solution of a problem. If the feasible solution of a problem can be defined as a set of positive real numbers *R* ^*n*+^ in *n*-dimensional space, then any vector *x*_*i*_ ∈ *R* ^*n*+^ with ${x}_i=\left\{{x}_i^1,{x}_i^2,\cdots, {x}_i^n\right\}$, *i* = 1 ,  ⋯  , *N*, is a valid molecule representing a valid solution of the problem and ${x}_i^k$, *k* = 1 ,  ⋯  , *n*, are thought of atoms representing the decision variables. The representation of molecule *x* ∈ *R* ^*n*+^ can be in the form of numbers, an array similar to chromosomes in genetic algorithms [23, 24], a matrix, or a graph similar to tree structure in genetic programming [25, 26]. A change in a molecule *x* ∈ *R* ^*n*+^ with higher energy state can only occur by means of chemical reaction. The chemical reaction changes the energy state of the molecule and results in a new molecule ${x}^{\prime}\in {R}^{n+}$ with a lower energy state. Therefore, minimisation of energy is considered as the objective in CRO and hence PE is defined as the objective function when evaluating a solution. PE is the energy responsible for a stable structure whereas KE is the energy needed for the movement of molecules and collision between molecules such that the reaction can happen. KE of the molecule helps it escape from the local minimum if the solution is stuck at a local minimum. If *x* represents the molecule with a certain structure (i.e. configuration of atoms), then an objective function, *f*(⋅), is defined as equal to PE:

20 $$ \mathrm{PE}(x)= f(x) $$

**Fig. 5:**
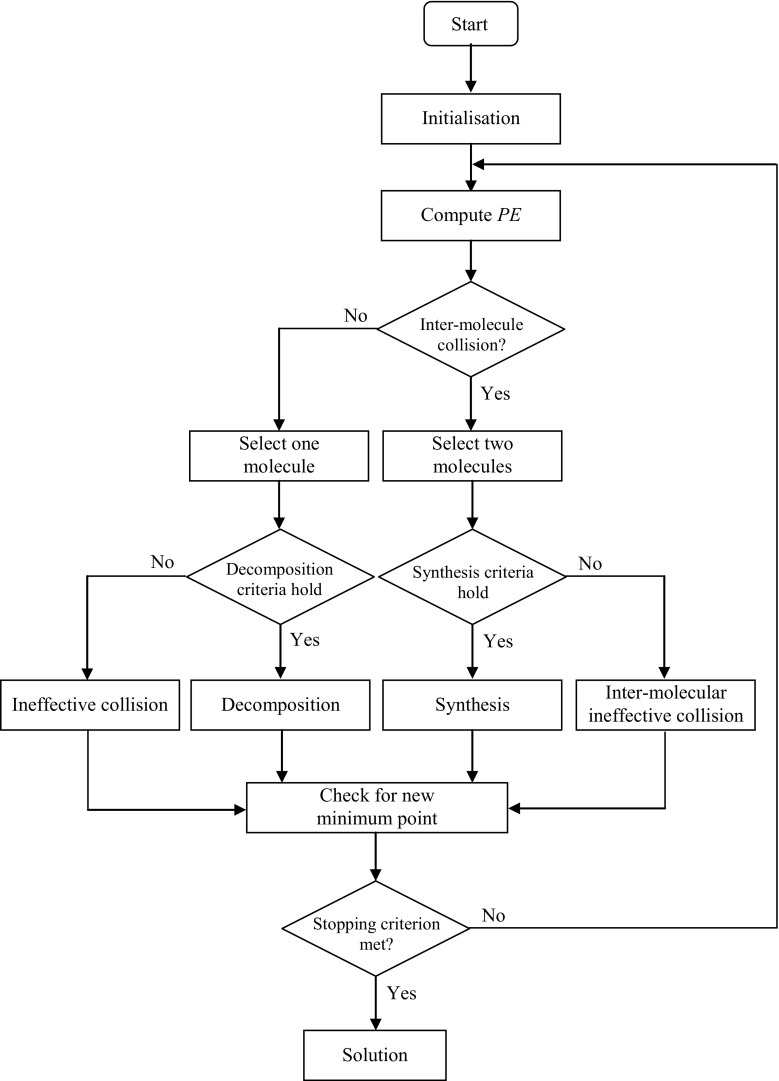
Flow diagram of CRO

Lam and Li [18] presented the CRO meta-heuristic algorithm inspired by chemical reactions. In CRO, decision variables are like atoms that form a molecule and a molecule is a representation of solution of a problem. A population of molecules is generated randomly within the search space. The molecules undergo chemical reaction-like transformation. Four types of chemical reaction operations are used: on-wall ineffective collision, decomposition, inter-molecular ineffective collision, and synthesis as discussed earlier. The parameter MoleColl decides on the fraction of all elementary reactions that involve more than one molecule, i.e. inter-molecular reactions in CRO. The process continues until a minimum of energy is reached as defined by the objective function, Eq. (20). This objective function is problem-dependent. The CRO algorithm is illustrated by the flow diagram in Fig. 5.

The operators of the CRO algorithm are the decomposition and synthesis operations as mechanisms for generating new solutions for exploring the search space, and the on-wall ineffective and inter-molecular ineffective collisions operations as the mechanisms for generating solutions from the neighbourhood structure for exploiting the search space. The decomposition and synthesis operators act as diversification and ineffective and inter-molecular ineffective collisions operators act as intensification for the algorithm [27].

There are mainly four parameters in CRO. These are population size, KE loss rate, fraction of uni-molecular reaction, and initial KE. Population size, denoted as PopSize, is the initial number of solutions generated randomly in the solution space. KE_LossRate_ is the loss rate of KE during reaction, i.e. it is the upper limit of percentage of KE lost to the environment during on-wall ineffective collisions. Fraction of uni-molecular reaction, denoted as MoleColl, is the fraction of molecules that undergo uni-molecular or inter-molecular reactions. If MoleColl is less than a random number, *ρ*, it will result in a uni-molecular collision. Otherwise, an inter-molecular collision will take place. It is to be noted that a uni-molecular collision will always take place when there remains only one molecule in the population. Initial KE, denoted as KE_0_, is the initial value assigned to each element of *KE* in the initialization stage.

## Variants of CRO

CRO algorithm is a recent addition to the meta-heuristic algorithm family. Researchers made simple modifications while applying the algorithm to different application domains which later became known as variants of CRO algorithms such as real-coded CRO, opposition-based CRO, and orthogonal CRO. Some researchers attempted to improve the operators borrowing ideas from other meta-heuristic algorithms such as differential evolution (DE) [28, 29] and particle swarm optimisation (PSO) [30, 31] which led to hybrid CRO algorithms. These variants are discussed briefly in the sequel.

### Real-Coded CRO (RCCRO)

The original version of CRO [18, 24] is designed for discrete optimisation problems. An extension of CRO algorithm is proposed for continuous problems by Lam et al. [32], which has become known as real-coded CRO. Three modifications are introduced: solution representation, neighbourhood operator, and boundary constraint handling.

A solution of an optimisation problem in standard CRO [18, 24] is represented by a molecular structure *x* = {*x*_1_, *x*_2_,  ⋯ , *x*_*n*_} where each individual *x*_*i*_, *i* = 1 , 2 ,  ⋯  , *n* can be of binary or integer type. In real-coded CRO, each *x*_*i*_ is implemented using a floating-point number. To deal with continuity, continuous search ability is incorporated into the neighbourhood search operator *N*(⋅). If the problem does not impose any constraints on relations between solution variables, *x*_*i*_ can be treated independently and a perturbation is defined as

21 $$ {x}_i^{\prime }= N\left({x}_i\right)={x}_i+{\delta}_i $$

where *δ*_*i*_ is a probabilistic perturbation in the *i*-th element, e.g. Gaussian, Cauchy, Lévy etc. For example, it may defined by Gaussian distribution of the form *δ*_*i*_ = *N*(*μ*, *σ* ^2^) with mean *μ* and variance *σ* ^2^.

The perturbation depends mainly on the starting point for the solution *x*_*i*_, direction from the mean *μ* and step-size based on the spread *σ*. In general *σ* is fixed in real-coded CRO during the execution of the algorithm. Too large or too small value for *σ* will make the algorithm inefficient. Lam et al. [32], therefore, proposed an adaptive scheme for *σ* where the initial value of *σ* is set equal to solution space (*u* − *l*) (upper bound minus lower bound values) and decreased gradually by a factor *θ*.

The perturbation of a solution *x*_*i*_ may go out of bound defined by the lower (*l*_*i*_)and upper (*u*_*i*_) bounds. The boundary constraints can be handled by bringing the solution *x*_*i*_ back within boundary [32]. A simple technique such as reflecting scheme, where *x*_*i*_ reflects back by the same amount from the boundary, can be used. ${x}_i^{\prime }$ is then defined by

22 $$ {x}_i^{\prime }=\left\{\begin{array}{l}2\times {l}_i-{x}_i\kern0.5em  if\kern0.1em {x}_i<{l}_i\;\hfill \\ {}\begin{array}{cc}\hfill 2\times {u}_i-{x}_i\hfill & \hfill if\kern0.1em {x}_i<{l}_i\hfill \end{array}\hfill \end{array}\right. $$

The effectiveness and performance of the real-coded CRO algorithm has been verified on a number of uni-modal, high-dimensional multi-modal, and low-dimensional multi-modal benchmark functions. Bhattacharjee et al. [33] applied real-coded CRO to minimisation of total power generation cost by scheduling different power plants for certain intervals of time.

### Opposition-Based CRO

Opposition-based approach in learning was proposed by Tizhoosh [34] with the assumption that if a set of randomly generated numbers can not satisfy a criterion, then the opposite set of numbers may a have higher possibly to satisfy the criterion. It is found that opposition-based approach improves computational efficiency. Many researchers have used opposition-based approach in optimisation problems [35–37] where they used the current population and its opposite population. Bhattacharjee et al. [38] used opposite and quasi-opposite numbers in one-dimensional space in the CRO algorithm. An opposite number $\overset{\smile }{x}$ of any real number *x* ∈ [*a*, *b*] is defined by

23 $$ \overset{\smile }{x}= a+ b- x $$

Bhattacharjee et al. [38] defined the quasi-opposite number ${\overset{\smile }{x}}_q$as

24 $$ {\overset{\smile }{x}}_q=\operatorname{rand}\left( c,\overset{\smile }{x}\right) $$

where *c* is the centre of the interval [*a*, *b*]. *c* can be estimated as the mean of the interval [*a*, *b*], i.e. *c* = (*a* + *b*)/2. Similarly, reflected quasi-opposite number ${\overset{\smile }{x}}_{qr}$ is defined by

25 $$ {\overset{\smile }{x}}_{qr}=\mathit{\operatorname{rand}}\left( c, x\right) $$

Bhattacharjee et al. [38] extended one-dimensional representation of *x* to two-dimensional representation. A population of molecular set is generated and then a quasi-opposite molecular matrix, denoted as QOM, is formed from the molecular set using a parameter *J*_*r*_ ∈ [0, 1] called jumping rate as follows:

If r*and* < *J*_*r*_

$$ QOM\left( i, j\right)=\mathit{\operatorname{rand}}\left( c,\overset{\smile }{x}\right)\ \mathrm{with}\  i=1,\cdots, PopSize\ \mathrm{and}\kern0.5em  j=1,\cdots, n $$

where *PopSize* is the population size and *n* is the number of variables in the optimisation problem.

The performance of the opposition-based real-coded CRO has been verified on short-term hydrothermal scheduling problem. The total minimum, maximum, and average system costs are obtained within 25 trials demonstrating the algorithm has good exploration and exploitation ability [38]. It is found that opposition-based approach helps improve convergence speed in optimisation algorithms.

### Orthogonal CRO

CRO algorithm traverses through search space in a random manner [39] which eventually limits the search scope and slows down the convergence speed. A meta-heuristic algorithm needs to explore promising regions. The exploration becomes difficult when an optimisation problem has a large number of decision variables to be optimised within a limited number of iterations. The orthogonal experimental design is an approach to find the best combination of different factors within a small number of trials. Such orthogonal array [40] has been applied in simulated annealing [41, 42], PSO [43], and genetic algorithm [44] with better results. Li et al. [45] proposed an orthogonal CRO algorithm by introducing a quantisation orthogonal crossover (QOX) operator, where the decision variables are quantised into different levels and the variables are divided into groups. The groups are treated as factors in orthogonal CRO. Then, an individual molecule is created using this information. The QOX operator is used for the synthesis operation in the CRO algorithm as follows:

If (*r* < MoleColl) Select randomly two molecules *x*_1_ , *x*_2_ from the population If (synthesis criteria hold) Create new molecule *x*^′^ using orthogonal crossover operation QOX(*x*_1_ , *x*_2_)

The molecules {*x*_1_ , *x*_2_} represent molecular structures described earlier. The effectiveness and performance of the orthogonal CRO have been verified on 23 well-known uni-modal, high-dimensional multi-modal, and low-dimensional multi-modal benchmark functions. The approach was showed to be less efficient for low-dimensional functions [45]. Duan and Gan [46] use an orthogonal multi-objective CRO for optimal design of a brushless DC motor.

### Adaptive Collision CRO

In the standard CRO, there is an overlap between functionalities of inter-molecular and on-wall ineffective collision operators leading to unnecessary computation times. To reduce this functional overlap, an adaptive collision scheme is introduced by Yu et al. [47, 48]. The adaptive collision consists of a new inter-molecular ineffective collision operator and an adaptive collision scheme.

In the inter-molecular ineffective collision operation, generally two on-wall ineffective collisions take place at the same time. Therefore, an inter-molecular operator is introduced to ineffective collisions such that it makes a difference between these two on-wall ineffective collisions. Two molecules {*x*_*i*_,*x*_*j*_} are randomly selected and their fitness values (i.e. PE values) are calculated. Let PE(*x*_*i*_)be greater than PE(*x*_*j*_). Based on the PE values, two approaches are deployed to modify the molecules instead of neighbourhood operation usually employed in on-wall ineffective collision [47, 48].

26 $$ \left\{\begin{array}{c}\hfill {x}_i^{\prime d}=\left({x}_i^d-{x}_j^d\right)\times {r}^d+{x}_i^d\hfill \\ {}\hfill {x}_j^{\prime d}=\left({x}_i^d-{x}_j^d\right)\times {r}^d+{x}_j^d\hfill \end{array}\right. $$

where ${x}_i^{\prime d}$ and ${x}_j^{\prime d}$ are the new molecules of the *d*-th element, ${x}_i^d$ and ${x}_j^d$ are old molecules of the *d*-th element and *r* ^*d*^ is a random number for each element over the interval [0, 1]. The mechanism expressed by Eq. (26) ensures that the new molecules ${x}_i^{\prime d}$ and ${x}_j^{\prime d}$ are not similar.

In the adaptive collision scheme, an adaptive collision rate (CollRate) is introduced. The CollRate is the ratio of occurrence of on-wall ineffective collision and inter-molecular reaction. The CollRate plays a critical role in the CRO performance. In standard CRO, CollRate is a user-defined parameter, which is fixed during execution and usually chosen empirically by users [49]. In adaptive collision scheme, CollRate is defined as a sigmoid function [47, 48] with two parameters count and FE_max_.

27 $$ \mathrm{CollRate}=\frac{1}{1+ \exp \left[-6\times \frac{\mathrm{count}}{{\mathrm{FE}}_{\max }}\right]} $$

The parameter count is the number of successful inter-molecular reaction. The value of count is incremented when a successful inter-molecular reaction occurs and it is decremented when an on-wall collision occurs. The parameter FE_max_ is the maximum allowable value for function evaluations. The performance of the adaptive CRO has been verified experimentally on 16 different benchmark functions and compared with standard CRO [47, 48].

### Elitist CRO

In the standard CRO algorithm, molecules are selected randomly. Though the random selection contributes to the diversity of the population, it impacts on the convergence rate. Duan and Gan [50] proposed an elitist CRO (ECRO) algorithm by introducing elitist strategies for selection, evolution, and crossover. Two new attributes of the molecule are introduced in ECRO: affinity and concentration. The affinity and concentration identify the quality of solution and similarity between solutions respectively. The efficiency of the ECRO has been verified on a contour-based target recognition problem.

### Hybrid CRO and DE

Roy et al. [51] propose an improvement to CRO algorithm by introducing the mutation and crossover operators borrowed from Differential Evolution (DE) algorithm [52] and called it hybrid DE-CRO. The CRO operators such as on-wall ineffective collision operation, decompose operation, inter-molecular ineffective collision operation and synthesis operation are implemented using mutation and crossover operation of DE.

The on-wall ineffective collision operation in CRO is implemented using the mutation operation of DE. A new molecule is generated using the mutation operation as follows

28 $$ {x}_{ij}^{\prime }={x}_{ij}+{F}^{\ast}\left({x}_{mj}-{x}_{nj}\right) $$

where ${x}_{ij}^{\prime }$ is the new *j*-th component of the i-th molecule, {*x*_*ij*_, *x*_*mj*_, *x*_*nj*_} are the *j*-th components of three different molecules chosen randomly from the current population and *F* is a positive control parameter.

The decompose operation is implemented using the crossover operation. To perform the crossover, one molecule *x*_*m*_ is selected randomly from the population and another molecule *x*_*n*_ is generated randomly. Two new molecules ${x}_m^{\prime }$ and ${x}_n^{\prime }$ are created by applying crossover operation on *x*_*m*_ and *x*_*n*_.

The inter-molecular ineffective collision operation is implemented using crossover operation. Two new molecules are created by performing crossover operation on two randomly selected molecules *x*_*m*_ and *x*_*n*_ from the population.

Molecules are modified using synthesis collision operation implemented by applying conventional crossover operation from genetic algorithm.

The effectiveness and performance of the DE-CRO algorithm has been verified on four test systems of conventional static economic load dispatch problem [51]. Dutta et al. [53] applied the DE-CRO algorithm to unified power flow control problem to determine the optimal parameter setting for power system network.

### Hybrid CRO and PSO

There are some good features of PSO algorithm that can be incorporated into CRO for improving exploration or exploitation. Nguyen et al. [54] combined the explorative and exploitative features of PSO and CRO. Due to low efficiency, the decomposition and synthesis operations are eliminated and a PSO-based update operation is performed instead. New molecules are created using neighbouring operations of CRO and mechanisms of PSO described by Eqs. (29)–(30). These molecules can be considered as molecules of CRO or particles of PSO. PSO and CRO use the same population generated initially. The basic operations involved in PSO algorithm are described by

29 $$ {v}_i^d\left( k+1\right)= w\cdot {v}_i^d(k)+{c}_1{r}_1\left({p}_i^d-{x}_i^d\right)+{c}_2{r}_2\left({p}_{gi}^d-{x}_i^d\right) $$

30 $$ {x}_i^d\left( k+1\right)={x}_i^d(k)+{v}_i^d\left( k+1\right)\times T $$

where ${v}_i^d$ is the velocity of *i*-th particle, ${x}_i^d$, ${p}_i^d$, and ${p}_{gi}^d$ are the position, iteration best, and global best position of the *d*-th element respectively, *w* is the inertia weight, *c*_1_ and *c*_2_ are cognitive and social coefficient respectively, *r*_1_ and *r*_2_ are random numbers generated between [0, 1] and *T* is the time, which is unity to convert the velocity into position. The computation of molecules (i.e. position of particles) is straightforward. If a PSO update criterion is satisfied, then molecules are updated using the PSO algorithm, i.e. using Eqs. (29)–(30), otherwise inter-molecular ineffective collision operation and on-wall ineffective collision operation are performed. Thus, the hybrid CRO-PSO algorithm repeats the PSO update, inter-molecular ineffective collision operation and on-wall ineffective collision operation until termination condition is satisfied. The CRO-PSO algorithm has been applied to well-known uni-modal and multi-modal benchmark functions.

Zhang and Duan [55] proposed another version of hybrid PSO-CRO approach, called PCRO, for solving the image matching problem where the best molecule is saved in each iteration. Once the on-wall ineffective collision and inter-molecular ineffective collision are performed, the molecule is updated by the distance between the original molecule and the current best molecule. In PCRO, the PSO update mechanism is simplified.

31 $$ {v}_i^d\left( k+1\right)={c}_1{r}_1\left({p}_i^d-{x}_i^d\right)/ T $$

32 $$ {x}_i^d\left( k+1\right)={x}_i^d(k)+{v}_i^d\left( k+1\right)\times T $$

where ${v}_i^d$, ${x}_i^d$, and ${p}_i^d$ are the velocity, position, and iteration best position in the *d*-th element of the *i*-th particle, respectively. Li et al. [56] use a hybrid PSO-CRO algorithm for multi-object optimisation problems. The proposed algorithm balances the operators of CRO and PSO while exploring the search space effectively. The hybrid approach also improves convergence.

### Other Chemistry-Based Algorithms

There are a few other chemistry-inspired algorithms reported in the literature. They are artificial chemical process algorithm (ACPA) based on the principles of artificial chemical process [57], artificial chemical reaction optimization (ACRO) [58] based on a different set of bi-molecular and uni-molecular chemical reactions different from CRO including redox (reduction**-**oxidation) reactions, chemical reaction algorithm (CRA) based on the principles of artificial chemistry [59], and Gases Brownian motion optimisation (GBMO) algorithm based on the laws of Brownian motion and turbulent rotation motion of gas molecules [60].

## Conclusions

CRO algorithm is a recent addition to meta-heuristic family and to cognitive computation paradigm. The significant contribution of this paper is the introduction of inspiration from chemistry and the development of meta-heuristic algorithms based on the principles of chemical reactions, which are complementary to biology and physics inspired algorithms. CRO algorithm has attracted the attention of machine learning and cognitive computation community over the past few years and has been successfully applied in several real-world optimization problems.

To improve the performance of CRO and solution quality, a number of hybrid variants have been proposed. There has been little theoretical analysis done so far apart from the convergence analysis by Lam et al. [39]. Premature convergence, convergence speed, searching behaviours, and parameter selection are important issues in CRO that need further research.

## Footnotes

1 Recombination or crossover operation in EA generally selects two solutions randomly from the population and combines them to produce one or two offspring by choosing individual elements from each of them. A detailed account of discussion on EA crossover operations can be found in [20].
